# EHMTI-0330. The prevalence of undifferentiated connective tissue dysplasia syndrome in children with migraine

**DOI:** 10.1186/1129-2377-15-S1-D44

**Published:** 2014-09-18

**Authors:** Y Nesterovskiy, N Zavadenko

**Affiliations:** 1 Neurology Neurosurgery and Medical Genetics Department of Pediatric Faculty, N.I. Pirogov’s Russian National Research Medical University, Moscow, Russia

## Background

The efficiency of migraine therapy depends on it's co-morbidities. One of these co-morbidities is represented by undifferentiated connective tissue dysplasia (UCTD). In general population the joint hypermobility syndrome (JHS) may be present in 5-30% (Murray K.J., 2001) and minor congenital heart defects (MCHDs) in 5-35% (Freed L.A., 1999). In adults approximately 75% of patients with JHM suffer migraine (Bendik E.M., 2011).

## Aim

to study the manifestations of JHS and MCHDs in children and adolescents with migraine compared with their peers suffering tension-type headache (TTH).

## Method

109 children and adolescents with migraine with/without aura established by the diagnostic criteria of ICHD-2 were included into the study. The control group included 50 patients with TTH. The JHS was confirmed according to P.H. Beighton's scored assessment (1999). MCHDs were diagnosed by the heart ultrasound examination.

## Results

The signs of UCTD were revealed in 76 (69%) of patients with migraine. JHS was confirmed in 68 (62%). MCHDs were found in 42 (38%).

In TTH patients the manifestations of UCTD were noted in only 21 (42%) of cases in the form of JHS and in 9 (18%) as MCHDs, which rates are similar to the literature data for general population.

## Conclusion

High prevalence of UCTD manifestations in child and adolescent migraine patients can influence the frequency and severity of migraine attacks and worsen the patients' somatic status which may have the negative influence on their quality of life.

No conflict of interest.

